# Serial cell culture passaging *in vitro* led to complete attenuation and changes in the characteristic features of a virulent porcine deltacoronavirus strain

**DOI:** 10.1128/jvi.00645-24

**Published:** 2024-07-16

**Authors:** Liping Zhang, Ruiming Yu, Lianshun Wang, Zhongwang Zhang, Yanzhen Lu, Peng Zhou, Yonglu Wang, Huichen Guo, Li Pan, Xinsheng Liu

**Affiliations:** 1 State Key Laboratory for Animal Disease Control and Prevention, Lanzhou Veterinary Research Institute, Chinese Academy of Agricultural Sciences, Lanzhou, China; 2 Gansu Province Research Center for Basic Disciplines of Pathogen Biology, Lanzhou Veterinary Research Institute, Chinese Academy of Agricultural Sciences, Lanzhou, China; 3 Department of Disease and Biosafety Control, National Center of Technology Innovation for Pigs, Lanzhou, China; The Ohio State University, Columbus, Ohio, USA

**Keywords:** porcine deltacoronavirus, passage, attenuation, pigs, pathogenicity

## Abstract

Porcine deltacoronavirus (PDCoV) is an important enteric coronavirus that has caused enormous economic losses in the pig industry worldwide. However, no commercial vaccine is currently available. Therefore, developing a safe and efficacious live-attenuated vaccine candidate is urgently needed. In this study, the PDCoV strain CH/XJYN/2016 was continuously passaged in LLC-PK cells until passage 240, and the virus growth kinetics in cell culture, pathogenicity in neonatal piglets, transcriptome differences after LLC-PK infection, changes in the functional characteristics of the spike (S) protein in the high- and low-passage strains, genetic variation of the virus genome, resistance to pepsin and acid, and protective effects of this strain when used as a live-attenuated vaccine were examined. The results of animal experiments demonstrated that the virulent PDCoV strain CH/XJYN/2016 was completely attenuated and not pathogenic in piglets following serial cell passage. Genome sequence analysis showed that amino acid mutations in nonstructural proteins were mainly concentrated in Nsp3, structural protein mutations were mainly concentrated in the S protein, and the N, M, and E genes were conserved. Transcriptome comparison revealed that compared with negative control cells, P10-infected LLC-PK cells had the most differentially expressed genes (DEGs), while P0 and P240 had the least number of DEGs. Analysis of trypsin dependence and related structural differences revealed that the P10 S protein interacted more strongly with trypsin and that the P120 S protein interacted more strongly with the APN receptor. Moreover, the infectivity of P240 was not affected by pepsin but was significantly decreased after exposure to low pH. Furthermore, the P240-based live-attenuated vaccine provided complete protection to piglets against the challenge of virulent PDCoV. In conclusion, we showed that a PDCoV strain was completely attenuated through serial passaging *in vitro*. These results provide insights into the potential molecular mechanisms of PDCoV attenuation and the development of a promising live-attenuated PDCoV vaccine.

**IMPORTANCE:**

Porcine deltacoronavirus (PDCoV) is one of the most important enteropathogenic pathogens that cause diarrhea in pigs of various ages, especially in suckling piglets, and causes enormous economic losses in the global commercial pork industry. There are currently no effective measures to prevent and control PDCoV. As reported in previous porcine epidemic diarrhea virus (PEDV) and transmissible gastroenteritis virus studies, inactivated vaccines usually elicit less robust protective immune responses than live-attenuated vaccines in native sows. Therefore, identifying potential attenuation mechanisms, gene evolution, pathogenicity differences during PDCoV passaging, and immunogenicity as live-attenuated vaccines is important for elucidating the mechanism of attenuation and developing safe and effective vaccines for virulent PDCoV strains. In this study, we demonstrated that the virulence of the PDCoV strain CH/XJYN/2016 was completely attenuated following serial cell passaging *in vitro*, and changes in the biological characteristics and protection efficacy of the strain were evaluated. Our results help elucidate the mechanism of PDCoV attenuation and support the development of appropriate designs for the study of live PDCoV vaccines.

## INTRODUCTION

Porcine deltacoronavirus (PDCoV) is a novel porcine enteric coronavirus that is a member of the order Nidovirales, the family Coronaviridae, and the genus *Deltacoronavirus* (1). PDCoV can infect pigs of different ages, among which suckling piglets are the most susceptible. The main clinical symptoms are listlessness, loss of appetite, vomiting, persistent diarrhea, and severe dehydration (2, 3). Similar to porcine epidemic diarrhea virus (PEDV) and porcine transmissible gastroenteritis virus (TGEV), PDCoV mainly infects pig small intestinal epithelial cells and causes significant villous atrophy (4). However, compared with those caused by PEDV and TGEV, the severity of clinical symptoms and mortality (approximately 40%–80%) caused by PDCoV in sucking piglets are relatively low (5).

PDCoV was first detected in pig rectal swabs in Hong Kong in 2012. PDCoV broke out on pig farms in Ohio, USA, in early 2014 and subsequently spread to pig farms in Canada, South Korea, Thailand, Laos, Japan, Vietnam, Mexico, and mainland China (4, 6, 7). Recent studies have shown that in addition to infecting pigs, PDCoV can also infect calves and poultry and can cause diarrhea in poultry (8). Additionally, in 2021, Lednicky et al. isolated PDCoV strains from blood samples of three Haitian children with acute febrile illness, indicating that PDCoV has intrinsic potential for cross-species transmission and is a potential high-risk novel coronavirus pathogen (9).

PDCoV is an enveloped, positive-sense RNA virus. The full-length genome is approximately 25.4 kb long and contains 5′ and 3′ untranslated regions and at least seven open reading frames that encode polymerase protein 1a/1b, spike (S), envelope (E), membrane (M), nucleocapsid (N), nonstructural protein 6 (NS6), and nonstructural protein 7 (NS7) (10, 11). Among them, the S protein plays an important role in the fusion of viral and cell receptors and the induction of the body’s production of neutralizing antibodies, and the S protein is the main antigenic protein with high immunogenicity (12–14).

Because prevention and control strategies for PEDV and TGEV rely on the “gut–breast–sIgA axis,” it is speculated that this pathway is also suitable for controlling PDCoV (15). Compared with inactivated vaccines, live-attenuated vaccines are better at activating the “gut–breast–sIgA axis” pathway and inducing high levels of sIgA antibodies (16). However, there is currently no commercial vaccine against PDCoV. Therefore, it is particularly urgent to obtain a weakened PDCoV strain and further investigate the genetic variation and biological characteristics of attenuated PDCoV strains during *in vitro* passaging for the development of live PDCoV vaccines.

In this study, (i) the PDCoV CH/XJYN/2016 strain (GenBank accession number: MN064712) was serially passaged in LLC-PK cells to the 240th generation (P240), and the biological characteristics and pathogenicity of the P0, P10, P50, P100, P160, and P240 strains were studied; (ii) amino acid mutations in the PDCoV genome from P0 to P240 were compared and analyzed; (iii) transcription was compared between high- and low-passage LLC-PK cells after infection with P0, P10, and P240; (iv) using molecular docking and molecular dynamics simulation methods, the molecular recognition process of trypsin with the P10 and P120 S proteins was investigated, and the structural basis of the difference in trypsin dependence between high- and low-passage cells was preliminarily analyzed; (v) the resistance of P10 and P240 to pepsin and acid was determined; and (iv) the protection efficiency of the P240-based live-attenuated vaccine was determined. In summary, we obtained a completely attenuated PDCoV strain through *in vitro* serial passage. The differences in biological characteristics between the high and low generations of this strain were further clarified at the cellular, transcriptional, molecular structure, and animal pathogenicity levels. Moreover, the P240-based live-attenuated vaccine provided complete protection to piglets against virulent PDCoV challenge. This study identified candidate strains for the future development of live PDCoV vaccines and provides new ideas for studying the attenuation mechanism of PDCoV.

## RESULTS

### The adaptability of PDCoV strains during serial passage of LLC-PK cells gradually increased

**Fig 1:**
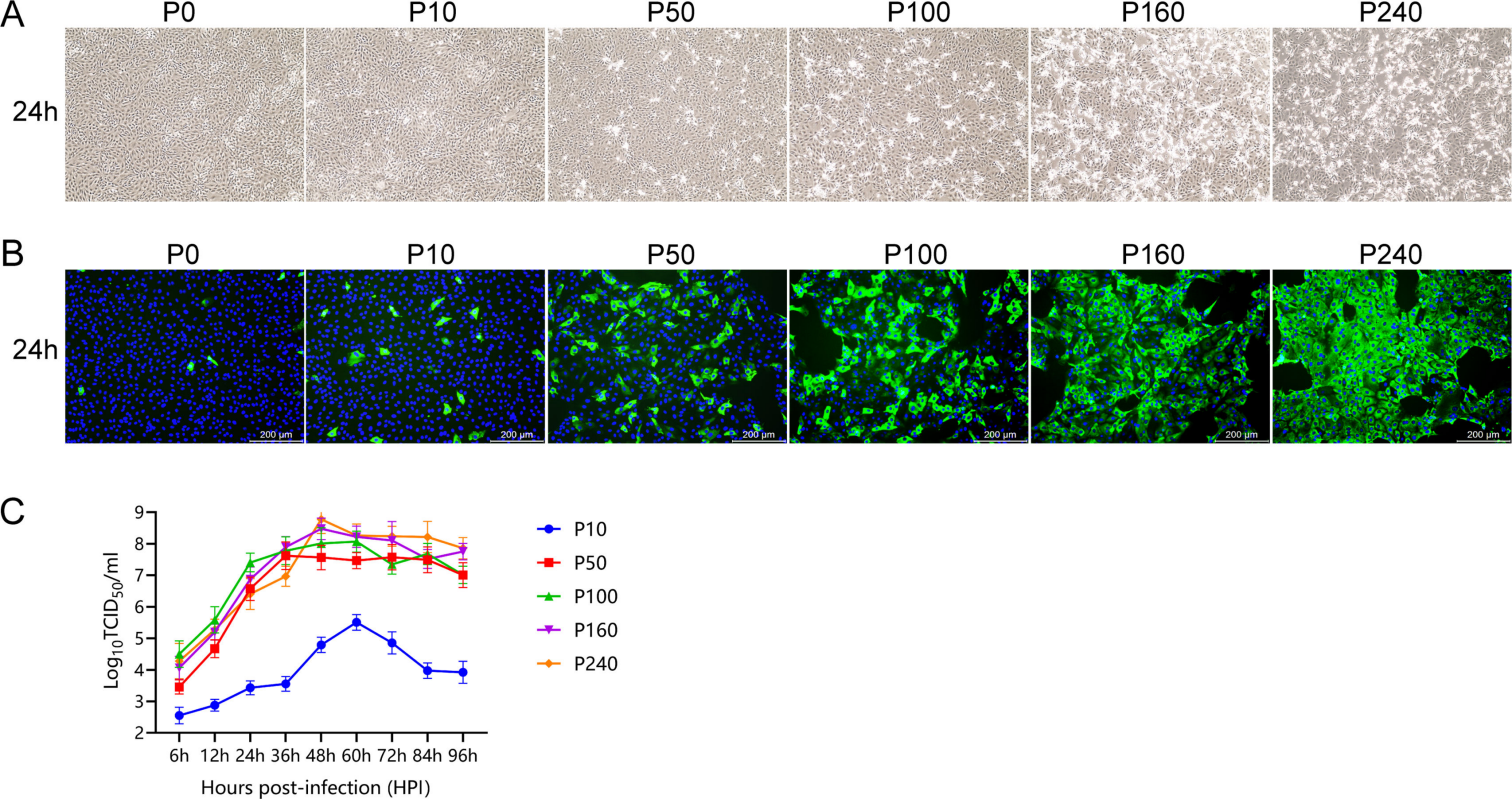
Biological characteristics of PDCoV CH/XJYN/2016 continuously passaged *in vitro*. (**A**) Cytopathic effects were observed at 24 h after LLC-PK cells were infected with P0, P10, P50, P100, P160, or P240 at an MOI of 0.01. (**B**) LLC-PK cells inoculated with P0, P10, P50, P100, P160, or P240 at an MOI of 0.01 were subjected to immunofluorescence using an Mab against the PDCoV N protein at 24 h. (**C**) The cell lysates were sampled at the designated time points and titrated using a 50% tissue culture infectious dose (TCID_50_) infectivity assay. LLC-PK cells were inoculated with P10, P50, P100, P160, or P240 at an MOI of 0.001.

LLC-PK cells were inoculated with the P0, P10, P50, P100, P160, or P240 strain to analyze the changes in the biological characteristics of the PDCoV CH/XJYN/2016 strain after serial passage *in vitro*. At 24 h after PDCoV infection at an multiplicity of infection (MOI) = 0.01, typical cytopathic effects (CPEs), e.g., rounded, aggregated, and detached cells, were observed (Fig. 1A). Specific green fluorescence upon treatment with a monoclonal antibody to the PDCoV N protein could be observed at 24 h after PDCoV infection at an MOI = 0.01 (Fig. 1B). Compared with the low-passage variants (P10), the high-passage variants (P50, P100, P160, or P240) produced more obvious CPEs and displayed richer specific green fluorescence (Fig. 1A and B). However, the P0 strain resulted in no visible CPEs in LLC-PK cells, only specific green fluorescence (Fig. 1A and B). These findings indicated that the P0 strain replicated in cells but had not completely acclimated to the new host cells. Growth kinetic curves of PDCoV-infected LLC-PK cells at an MOI = 0.001 showed that the viral titer of P10 peaked at 60 h, that of P50 peaked at 36 h, that of P100 peaked at 48–60 h, and that of P160 and P240 peaked at 48 h (Fig. 1C). Overall, these results indicated that the sensitivity and adaptability of the PDCoV CH/XJYN/2016 strain toward LLC-PK cells gradually increased during serial passage *in vitro*.

### The pathogenicity of PDCoV serial passage strains in 4-day-old piglets was weakened, or the strains were nonpathogenic

The clinical symptoms and fecal virus shedding of PDCoV P0-, P10-, P50-, P100-, P160-, and P240-infected 4-day-old piglets at 0–7 days post-inoculation (dpi) are shown in Table 1. Pasty or semifluid diarrhea was observed in two piglets in the P0 group (G1) at 1 dpi [fecal consistency (FC) score = 1.00 ± 1.41, mean cycle threshold (CT) value = 29.15 ± 2.53], and all five piglets had semifluid or liquid diarrhea at 2–3 dpi, in which higher viral RNA titers were detected at 3 dpi (FC score = 21.55 ± 3.00, CT value = 2.80 ± 0.45), and diarrhea symptoms were reduced at 3–7 dpi; however, two piglets still experienced pasty diarrhea with a small amount of viral RNA excretion at 7 dpi (FC score = 31.62 ± 2.45, CT value = 0.40 ± 0.55). One piglet in the P10 group (G2) had pasty diarrhea at 1 dpi (FC score = 0.60 ± 0.89, CT value = 30.93 ± 1.44), diarrhea worsened at 2–5 dpi, and five piglets had diarrhea at 3 dpi and higher viral RNA titers at 4 dpi (FC score = 2.60 ± 0.55, CT value = 23.53 ± 5.85). At 7 dpi in the P10 group, all the piglets had recovered with an increase in appetite (FC score = 0.20 ± 0.45, CT values: 32.83 ± 0.5). Diarrhea was observed in two piglets in the P50 group (G3) at 2 dpi (FC score = 1.00 ± 1.41, CT value = 27.42 ± 4.12), and the number of piglets with diarrhea reached four at 5 dpi (FC score = 1.40 ± 1.14, CT value = 27.62 ± 2.89). Then, the animals gradually recovered until one piglet experienced diarrhea at 7 dpi. None of the piglets in the P100 group (G4) had diarrhea (FC score = 0), but different titers of viral RNA were detected from 1 to 7 dpi (CT values: 23.65 ± 2.97–29.86 ± 1.19). None of the piglets in the P160 group (G5) had diarrhea (FC score = 0), but lower titers of viral RNA in feces were detected at 4–7 dpi (CT values: 30.76 ± 1.93–31.53 ± 1.78). No diarrhea symptoms were observed in any of the piglets in the P240 inoculation group (G6) at 0–7 dpi (FC score = 0), and limited viral RNA in feces was detected (CT values > 30). Piglets in the mock group did not have diarrhea during the entire experiment, and no fecal RNA shedding was detected.

**TABLE 1:** Clinical signs and fecal virus shedding of 4-day-old piglets inoculated with selected passages of the PDCoV CH/XJYN/2016 strain (P0, P10, P50, P100, P160, and P240)^*f*^

Groups	G1			G2			G3			G4			G5			G6			Mock		
Inoculation [no. of pigs, age (days)]	*n* = 5, 4			*n* = 5, 4			*n* = 5, 4			*n* = 5, 4			*n* = 5, 4			*n* = 5, 4			*n* = 3, 4		
Inoculum^*a*^	P0			P10			P50			P100			P160			P240			MEM		
Calculated inoculum infectious titer (log_10_TCID_50_/mL)^*b*^	–			4.6			7.4			8.3			8.4			8.5			–		
dpi	CT^*c*^	NP	FC^*d*^	CT	NP	FC	CT	NP	FC	CT	NP	FC	CT	NP	FC	CT	NP	FC	CT	NP	FC
0	33.45 ± 1.29	0/5	0	32.59 ± 0.79	0/5	0	33.50 ± 1.12	0/5	0	31.59 ± 1.09	0/5	0	31.32 ± 0.55	0/5	0	35.01 ± 1.19	0/5	0	–^*e*^	0/3	0
1	29.15 ± 2.53	2/5	1.00 ± 1.41	30.93 ± 1.44	1/5	0.60 ± 0.89	30.59 ± 0.91	0/5	0	29.86 ± 1.19			31.94 ± 1.24			35.60 ± 1.03					
2	25.72 ± 3.39	5/5	2.20 ± 0.84	18.69 ± 3.35	4/5	1.80 ± 1.30	27.42 ± 4.12	2/5	1.00 ± 1.41	23.65 ± 2.97			32.07 ± 1.20			30.82 ± 0.43					
3	21.55 ± 3.00	5/5	2.80 ± 0.45	25.64 ± 6.13	5/5	1.60 ± 1.52	27.14 ± 3.38	3/5	1.60 ± 1.52	25.21 ± 1.32			32.86 ± 1.19			30.72 ± 0.39					
4	26.30 ± 2.36	4/5	2.20 ± 1.30	23.53 ± 5.85	4/5	2.60 ± 0.55	26.72 ± 3.49	3/5	1.00 ± 1.41	26.24 ± 2.43			31.39 ± 1.78			36.34 ± 1.06					
5	28.55 ± 2.80	3/5	1.20 ± 1.10	23.71 ± 5.54	4/5	2.20 ± 0.84	27.62 ± 2.89	4/5	1.40 ± 1.14	25.02 ± 3.79			31.53 ± 1.78			33.49 ± 1.77					
6	30.24 ± 2.24	3/5	0.60 ± 0.55	29.34 ± 3.75	2/5	1.00 ± 0.71	29.93 ± 2.36	1/5	0.20 ± 0.45	25.34 ± 1.50			30.76 ± 1.93			32.91 ± 0.45					
7	31.62 ± 2.45	2/5	0.40 ± 0.55	32.83 ± 0.50	0/5	0.20 ± 0.45	31.53 ± 1.54	1/5	0.20 ± 0.45	26.65 ± 2.46			30.74 ± 1.52			33.13 ± 1.42					

^*a*^ All pigs were inoculated orally with 1 mL MEM-treated P0, P10 at doses of 10^4.6^ TCID_50_/pig, P50 at doses of 10^7.4^ TCID_50_/pig, P100 at doses of 10^8.3^ TCID_50_/pig, P160 at doses of 10^8.4^ TCID_50_/pig, P240 at doses of 10^8.5^ TCID_50_/pig, and mock group with 1 mL of MEM.

^*b*^ Original titers of PDCoV CH/XJYN/2016 strain (P10, P50, P100, P160, and P240).

^*c*^ A cut-off point was set at 30; CT values greater than 30 were considered negative or below the detection limit of RT-qPCR.

^*d*^ Score: 0 = normal; 1 = pasty; 2 = semiliquid; 3 = liquid. A score greater than or equal to 1 was considered diarrhea.

^*e*^ Samples with no CT value (no PDCoV RNA detected) are indicated by “–”.

^*f*^ CT value: the mean cycle threshold value; dpi: days post-inoculation; FC: fecal consistency; NP: number of PDCoV-positive pigs.

### The jejunal tissue damage of 4-day-old piglets infected by PDCoV serial passage strains was reduced, or no damage was observed

**Fig 2:**
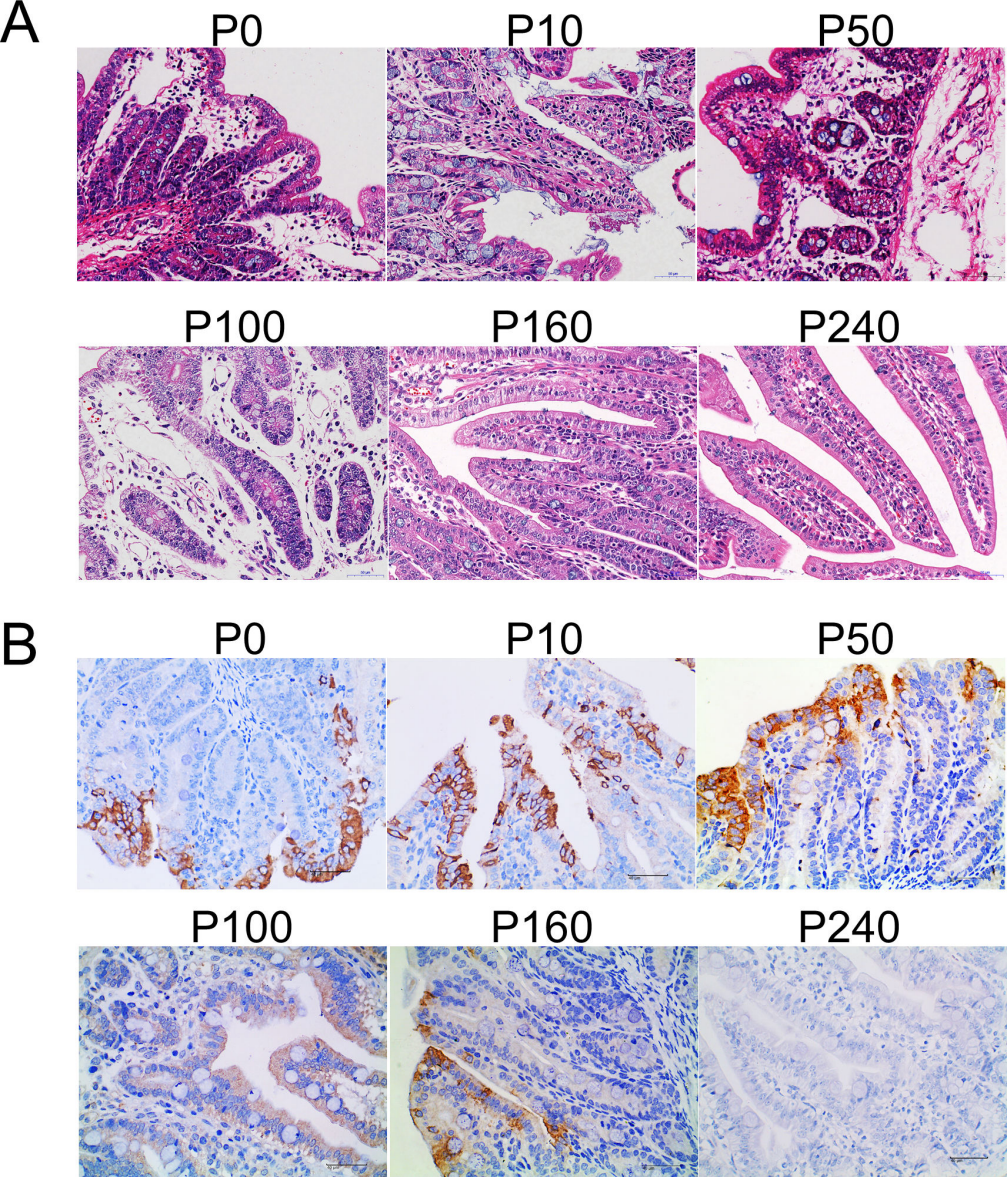
Histopathological and immunohistochemistry analyses of piglet samples inoculated with PDCoV CH/XJYN/2016-P0, P10, P50, P100, P160, or P240. (**A**) Villus shedding, submucosal edema, necrosis of the lamina propria, and increased lymphocytes and neutrophils were observed in jejunal intestines following infection with P0, P10, P50, and P100. No obvious pathological intestinal injury was observed in the P160 or P240 infection groups or the mock group. (**B**) PDCoV antigen signals are brown in color and were detected in jejunal intestinal epithelial cells infected with P0, P10, P50, and P100, and a small amount of PDCoV antigen was detected at P160. No antigen signal was observed in the P240 and mock groups.

After PDCoV P0, P10, P50, P100, P160, and P240 infection of 4-day-old piglets at 7 dpi, the piglets were dissected, and jejunal tissues were selected for histopathology and immunohistochemistry. Histopathological analysis revealed pathological injuries such as jejunal villus shedding, submucosal edema, necrosis of the lamina propria, and increases in lymphocyte and neutrophil counts in the P0, P10, P50, and P100 infection groups (Fig. 2A). No obvious pathological intestinal injury was observed in the P160 or P240 infection groups or the mock group (Fig. 2A). PDCoV antigens were detected in the jejunal intestinal epithelial cells of the P0-, P10-, P50-, P100-, and P160-infected groups, but the amount of PDCoV detected was significantly greater in the P0-, P10-, P50-, and P100-infected groups than in the P160-infected group (Fig. 2B). In contrast, no PDCoV antigen was detected in the P240-infected group or the mock group (Fig. 2B).

### The infectivity of PDCoV P240 was not affected by pepsin but significantly decreased after exposure to low pH

**Fig 3:**
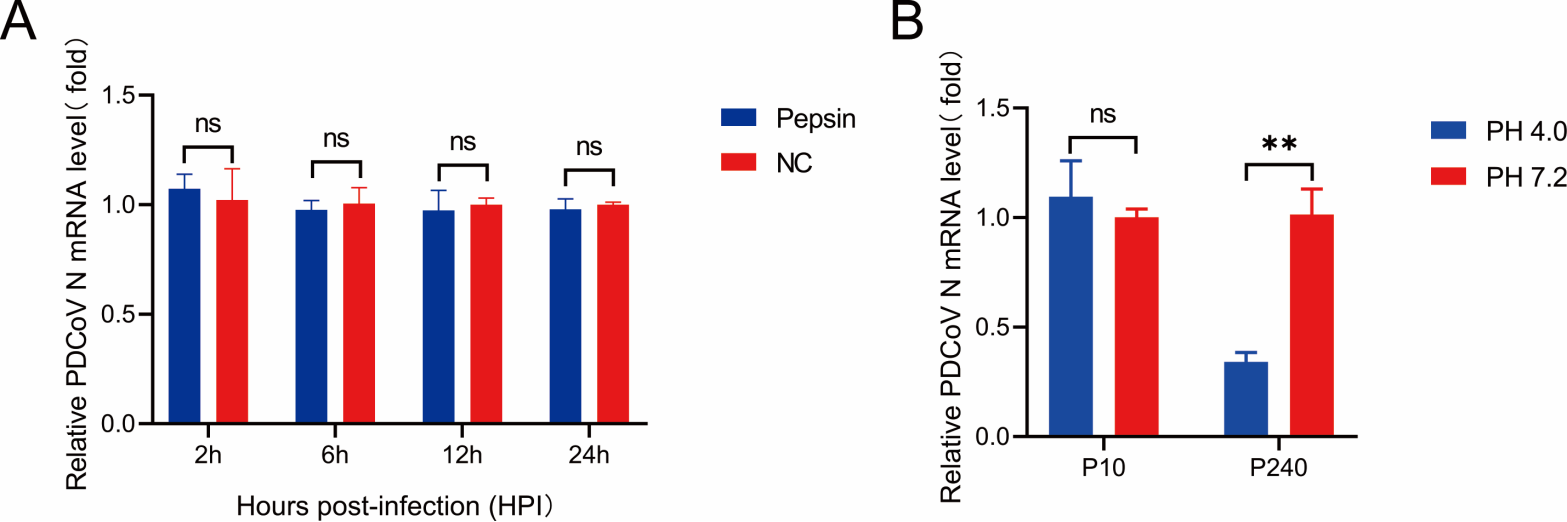
Effect of pepsin and acid on the infectivity of PDCoV P240 in LLC-PK cells. (**A**) PDCoV P240 virus was treated with pepsin for 60 min, after which the PK cells were infected at an MOI = 0.1. Cell samples were collected at 2, 6, 12, and 24 h for RT-qPCR detection. (**B**) P10 and P240 cells were treated with MEM at a pH of 4.0 (low) or 7.2 (normal) for 60 min and then used to infect LLC-PK cells at an MOI of 0.1; cell samples were collected at 12 h for RT-qPCR detection.

To verify whether infectivity is related to pepsin and pH tolerance, PDCoV P240 (MOI = 0.1) was treated separately with pepsin and exposed to different pH levels; then, the levels of replication of P240 in LLC-PK cells were determined by quantitative reverse transcription PCR (RT-qPCR). The results of pepsin tolerance assays showed that there was no significant difference in PDCoV mRNA levels between the pepsin treatment group and the control group at 2, 6, 12, and 24 h, indicating that the infectivity of P240 was not affected by pepsin (Fig. 3A). However, the results from different pH treatments showed that a low pH (pH 4.0) markedly decreased the mRNA level of P240 but had no significant effect on P10 (Fig. 3B), indicating that P240 cannot withstand the low pH environment of the gastric fluid of suckling piglets. This result suggested that the resistance of PDCoV to acid was correspondingly reduced following serial cell passage. Therefore, this reduction may be one of the reasons that high-passage PDCoV was attenuated in piglets. This result also explained why virulent or low-passage PDCoV could reach and infect intestinal epithelial cells through the stomach of piglets.

### Analysis of genomic variations in serially passaged strains of PDCoV

The genome sequences of P0–P240 were determined and analyzed to investigate the causes of the reduced pathogenicity of PDCoV CH/XJYN/2016 after continuous passage in LLC-PK cells. As shown in Tables 2 and 3, the amino acid mutations of nonstructural proteins were mainly concentrated in Nsp3, with five mutations. Amino acid mutations in structural proteins were mainly concentrated in the S protein, with 26 mutations, 16 in the S1 subunit (1–531 aa) and 10 in the S2 subunit (532–1,160 aa), and the N, M, and E genes were more conserved, with only one to two mutations. P20–P60 had a high frequency of S gene mutations, while P110–P130 and P150–P190 were stable, and no base mutations were detected. The 26 amino acid mutations were as follows: P20 had the most amino acid mutations, with five mutations (N162K, N169K, N397K, Q489L, and N1029K); P30 had three amino acid mutations (H168R, T183I, and A235V); P10 (T527A and T809I), P40 (I563L and I800T), P50 (I190V and Q614H), P60 (H140R and L563M), P100 (H100Q and L1095I), P230 (D284E and K940A), and P240 (H229Y and M884K) had two amino acid mutations; and P80 (L34R), P140 (T1147I), P150 (D139E), and P200 (D194H) each had one amino acid mutation.

**TABLE 2:** Amino acid changes of S proteins during continuous passage of PDCoV CH/XJYN/2016 in LLC-PK cells^*a*^

Amino acid position	S protein																								
Passages	34	100	139	140	162	168	169	183	190	194	229	235	284	397	489	527	563	614	800	809	884	940	1,029	1,095	1,147
P0	L	H	D	H	N	H	N	T	I	D	H	A	D	N	Q	T	I	Q	I	T	M	K	N	L	T
P10	L	H	D	H	N	H	N	T	I	D	H	A	D	N	Q	A	I	Q	I	I	M	K	N	L	T
P20	L	H	D	H	K	H	K	T	I	D	H	A	D	K	L	A	I	Q	I	I	M	K	K	L	T
P30	L	H	D	H	K	R	K	I	I	D	H	V	D	K	L	A	I	Q	I	I	M	K	K	L	T
P40	L	H	D	H	K	R	K	I	I	D	H	V	D	K	L	A	L	Q	T	I	M	K	K	L	T
P50	L	H	D	H	K	R	K	I	V	D	H	V	D	K	L	A	L	H	T	I	M	K	K	L	T
P60	L	H	D	R	K	R	K	I	V	D	H	V	D	K	L	A	M	H	T	I	M	K	K	L	T
P70	L	H	D	R	K	R	K	I	V	D	H	V	D	K	L	A	M	H	T	I	M	K	K	L	T
P80	R	H	D	R	K	R	K	I	V	D	H	V	D	K	L	A	M	H	T	I	M	K	K	L	T
P90	R	H	D	R	K	R	K	I	V	D	H	V	D	K	L	A	M	H	T	I	M	K	K	L	T
P100	R	Q	D	R	K	R	K	I	V	D	H	V	D	K	L	A	M	H	T	I	M	K	K	I	T
P110	R	Q	D	R	K	R	K	I	V	D	H	V	D	K	L	A	M	H	T	I	M	K	K	I	T
P120	R	Q	D	R	K	R	K	I	V	D	H	V	D	K	L	A	M	H	T	I	M	K	K	I	T
P130	R	Q	D	R	K	R	K	I	V	D	H	V	D	K	L	A	M	H	T	I	M	K	K	I	T
P140	R	Q	D	R	K	R	K	I	V	D	H	V	D	K	L	A	M	H	T	I	M	K	K	I	I
P150	R	Q	E	R	K	R	K	I	V	D	H	V	D	K	L	A	M	H	T	I	M	K	K	I	I
P160	R	Q	E	R	K	R	K	I	V	D	H	V	D	K	L	A	M	H	T	I	M	K	K	I	I
P170	R	Q	E	R	K	R	K	I	V	D	H	V	D	K	L	A	M	H	T	I	M	K	K	I	I
P180	R	Q	E	R	K	R	K	I	V	D	H	V	D	K	L	A	M	H	T	I	M	K	K	I	I
P190	R	Q	E	R	K	R	K	I	V	D	H	V	D	K	L	A	M	H	T	I	M	K	K	I	I
P200	R	Q	E	R	K	R	K	I	V	H	H	V	D	K	L	A	M	H	T	I	M	K	K	I	I
P210	R	Q	E	R	K	R	K	I	V	H	H	V	D	K	L	A	M	H	T	I	M	K	K	I	I
P220	R	Q	E	R	K	R	K	I	V	H	H	V	D	K	L	A	M	H	T	I	M	K	K	I	I
P230	R	Q	E	R	K	R	K	I	V	H	H	V	E	K	L	A	M	H	T	I	M	N	K	I	I
P240	R	Q	E	R	K	R	K	I	V	H	Y	V	E	K	L	A	M	H	T	I	K	N	K	I	I

^*a*^ Grey indicates that the amino acid at this site has mutated. Dark gray indicates that the amino acid at this site mutated for the second time in different generations.

**TABLE 3:** The mutations of amino acids except S proteins during the continuous passage of PDCoV CH/XJYN/2016 in LLC-PK cells^*a*^

	ORF1a										ORF1b				E	M		N	NS7	
Amino acid position	Nsp2	Nsp3					Nsp4	Nsp5	Nsp7		Nsp12			Nsp14						
Passages	131	828	983	1,297	1,549	1,589	2,200	2,682	3,134	3,165	116	228	673	1,872	14	73	157	329	94	161
P0	A	G	A	D	T	T	A	N	A	A	H	T	D	I	Y	I	P	E	L	G
P10	A	G	A	D	T	A	V	N	E	S	Y	T	Y	I	Y	I	P	E	P	G
P20	A	G	A	D	M	A	V	N	E	S	Y	T	Y	I	Y	I	P	E	P	E
P30	A	G	A	D	M	A	V	N	E	S	Y	T	Y	I	Y	I	S	E	P	E
P40	A	G	A	D	M	A	V	N	E	S	Y	T	Y	I	C	I	S	E	P	E
P50	A	G	A	D	M	A	V	N	E	S	Y	T	Y	I	C	I	S	E	P	E
P60	A	G	A	D	M	A	V	N	E	S	Y	T	Y	I	C	I	S	E	P	E
P70	A	G	A	D	M	A	V	N	E	S	Y	T	Y	I	C	I	S	E	P	E
P80	A	G	A	D	M	A	V	N	E	S	Y	S	Y	I	C	I	S	E	P	E
P90	A	G	A	D	M	A	V	N	E	S	Y	S	Y	I	C	I	S	E	P	E
P100	A	G	A	D	M	A	V	N	E	S	Y	S	Y	I	C	I	S	E	P	E
P110	A	G	A	D	M	A	V	N	E	S	Y	S	Y	I	C	I	S	E	P	E
P120	S	G	A	D	M	A	V	N	E	S	Y	S	Y	I	C	I	S	E	P	E
P130	S	G	A	D	M	A	V	N	E	S	Y	S	Y	T	C	S	S	E	P	E
P140–P210	S	G	E	D	M	A	V	N	E	S	Y	S	Y	T	C	S	S	E	P	E
P220	S	G	E	E	M	A	V	K	E	S	Y	S	Y	T	C	S	P	E	P	E
P230	S	G	E	E	M	A	V	K	E	S	Y	S	Y	T	C	S	P	D	P	E
P240	S	W	E	E	M	A	V	K	E	S	Y	S	Y	T	C	S	P	D	P	E

^*a*^ There are mutations detected in Nsp2, Nsp3, Nsp4, Nsp5, Nsp7, Nsp12, Nsp14, E, M, N, and NS7, but not from Nsp6, Nsp8, Nsp9, Nsp10, Nsp11, Nsp13, Nsp15, Nsp16, and NS6 proteins.

^*b*^ Grey indicates that the amino acid at this site has mutated. Dark gray indicates that the amino acid at this site mutated for the second time in different generations.

### Transcriptome analysis of PDCoV P0-, P10-, and P240-infected LLC-PK cells

#### Differential gene expression analysis

**Fig 4:**
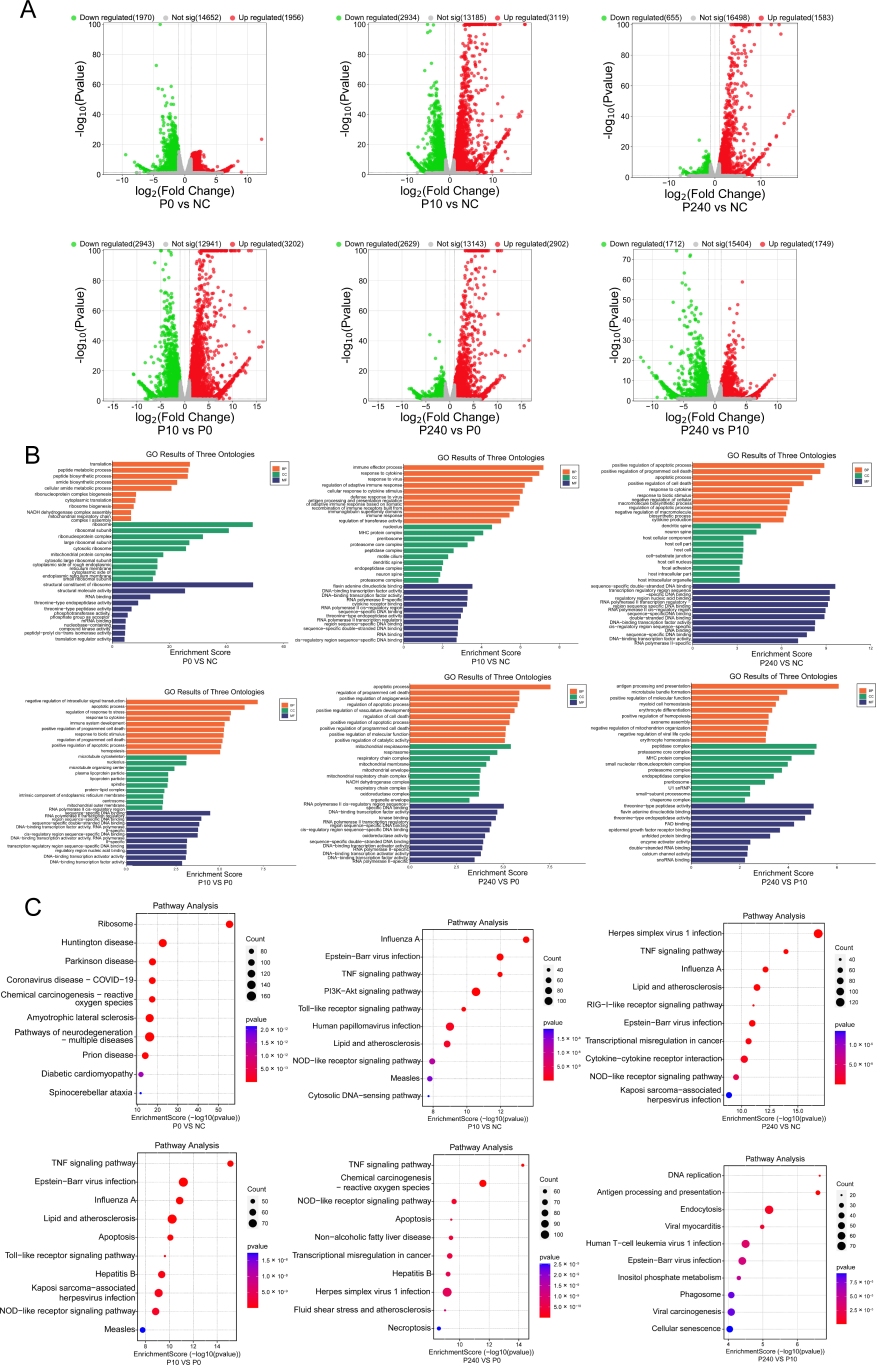
Transcriptome analysis of LLC-PK cells infected with the PDCoV CH/XJYN/2016 P0, P10, and P240 strains at an MOI of 0.1. (**A**) Volcano plot analysis of the DEGs in P0-, P10-, and P240-infected LLC-PK cells. (**B**) GO enrichment analysis of DEGs in LLC-PK cells in response to P0, P10, and P240 infection. (**C**) KEGG enrichment analysis of DEGs in LLC-PK cells in response to P0, P10, and P240 infection.

To further explore the molecular mechanisms underlying the differences in the pathogenicity and virulence of PDCoV serially passaged strains, we used transcriptome sequencing technology to perform differential gene expression analysis of LLC-PK cells infected with P0, P10, and P240 (Fig. 4A). In this study, *P*val ≤0.05 and |fold change| ≥ 2 were used as the criteria for screening DEGs. There were 3,926, 6,053, and 2,238 DEGs in P0, P10, and P240 compared with those in the negative control (NC) group, including 1,956, 3,119, and 1,583 upregulated genes and 1,970, 2,934, and 655 downregulated genes, respectively. Compared with those in the P0 group, there were 6,145 and 5,531 DEGs in P10 and P240, respectively, of which 3,202 and 2,902 genes were upregulated and 2,943 and 2,629 were downregulated. There were 3,461 DEGs between the P240 and P10 groups, of which 1,749 were upregulated and 1,712 were downregulated.

#### GO enrichment analysis of DEGs

To understand the biologically relevant functions of the DEGs, clusterProfiler software (17) was used for GO enrichment analysis. A *P*-adjust (FDR) <0.05 was used as the threshold for screening significant enrichment results. A GO term that satisfied these criteria was defined as a GO term that was significantly enriched in DEGs. The results are shown in Fig. 4B. Compared with those of the NC group, DEGs of P0, P10, and P240 were enriched in translation, ribosome, ribosomal structure, immune effect, cytokines, response to viruses, apoptosis, cytokine response, transcriptional regulation, and other biological processes. Compared with P0, P10 and P240 were more enriched in genes related to apoptosis, mitochondrial respiration, and transcriptional regulation. The DEGs of P240 were more enriched in molecular functions such as antigen processing and presentation, peptidase complex, proteasome complex, and threonine peptidase activity than were those of P10 (Fig. 4B).

#### KEGG enrichment analysis of DEGs

To determine the most important biochemical metabolic pathways and signal transduction pathways involving the DEGs, based on the differential expression analysis and KEGG annotation results, clusterProfiler software was used to identify significantly enriched KEGG pathways with *P*-adjust (false discovery rate, FDR) <0.05 as the threshold. The results showed that P0 DEGs were mainly enriched in the ribosome, Huntington’s disease, Parkinson’s disease, and coronavirus disease-COVID-19 signaling pathways. P10 DEGs were mainly enriched in the influenza A, Epstein‒Barr virus infection, TNF, P13K-Akt, and Toll-like receptor signaling pathways. P240 DEGs were mainly enriched in Herpes simplex virus 1 infection, influenza A, TNF, RIG-I-like receptor, and NOD-like receptor. Compared with P0, P10 DEGs were mainly enriched in TNF, Epstein‒Barr virus infection, influenza A, and other signaling pathways, while P240 DEGs were mainly enriched in TNF, chemical carcinogenesis reactive oxygen species, NOD-like receptors, and other signaling pathways. DEGs in P240 were more enriched in DNA replication, antigen processing and presentation, endocytosis, and other pathways than were those in P10 (Fig. 4C).

### Trypsin dependence test and structural analysis of the S protein

**Fig 5:**
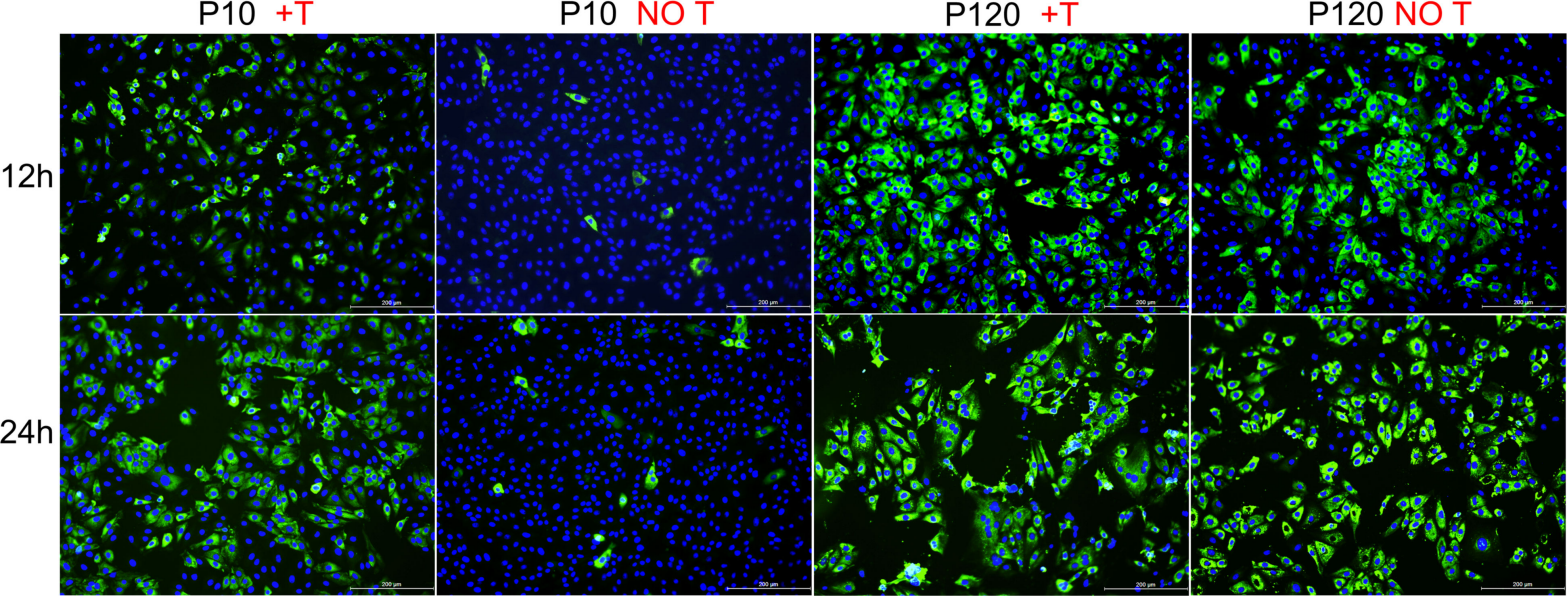
The proliferative characteristics of P10 and P120 in LLC-PK cells with or without trypsin. LLC-PK cells were infected with P10 or P120 at an MOI of 0.1 in the presence or absence of trypsin. Infected cells were fixed at 12 or 24 h post-infection and were then subjected to immunofluorescence staining for the nucleocapsid protein (green). Nuclei were stained with 4′,6-diamidino-2-phenylindole (DAPI) (blue). Bars, 200 µm.

Previous studies have shown that the isolation and culture of the PDCoV CH/XJYN/2016 strain require the addition of trypsin to the culture medium. In the attenuation process of the serial passaging of the PDCoV CH/XJYN/2016 strain in LLC-PK cells, the trypsin dependence of the PDCoV CH/XJYN/2016 strain gradually weakened as the number of passages increased until P120 when the strain was able to cause cytopathic effects and proliferate in LLC-PK cells without the addition of trypsin during the culture process. The IFA results are shown in Fig. 5. With the addition of trypsin and P10, after 12 h, 30% of the cells were infected with PDCoV, and at 24 h, 90% of the cells were infected with PDCoV. In contrast, in the absence of trypsin, only a few P10 cells were infected with PDCoV-P10 and did not proliferate. P120 infected LLC-PK cells well in the presence or absence of trypsin, and the cells could proliferate stably.

**Fig 6:**
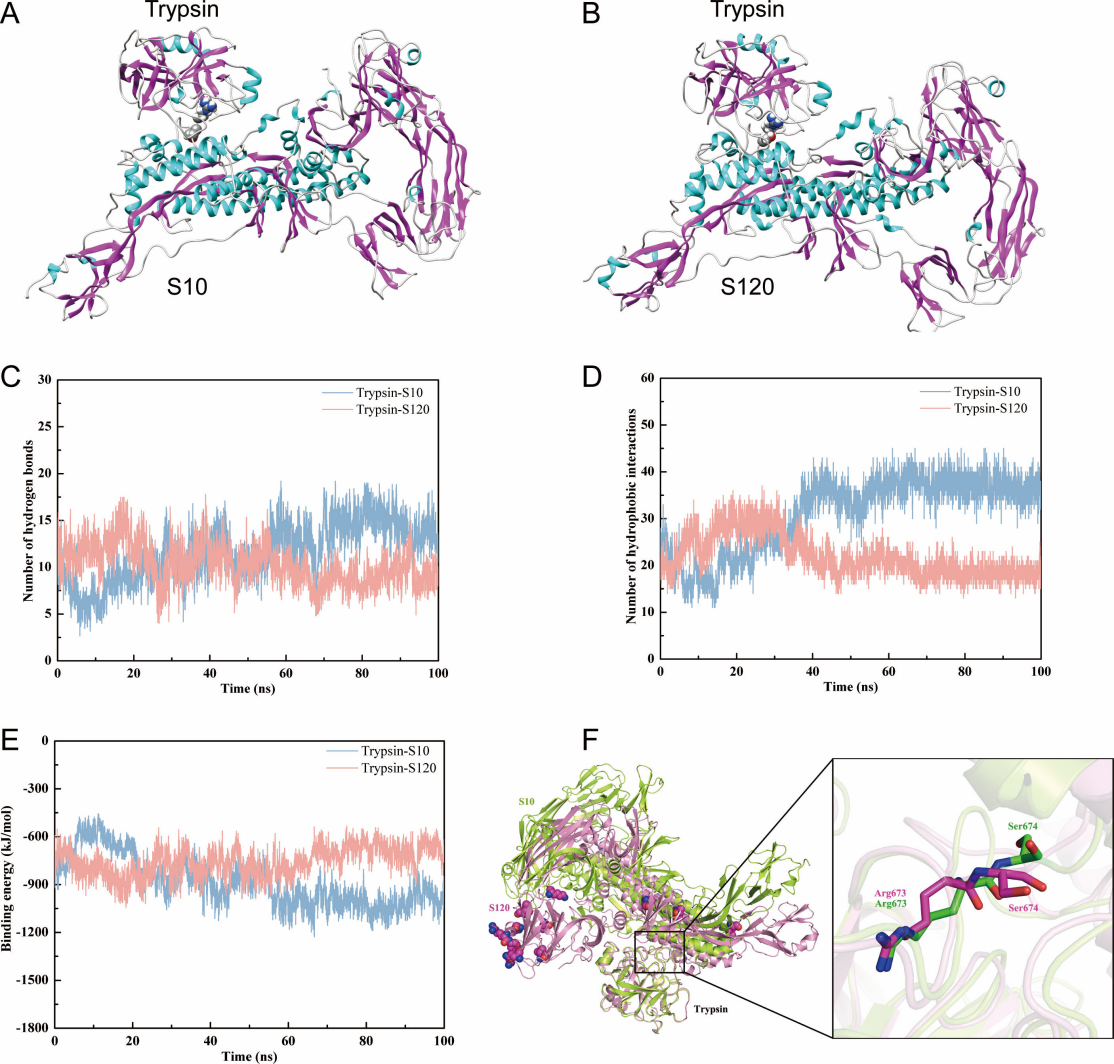
Molecular binding analysis of the S10/S120 (PDB: 6BFU) protein with trypsin (4AN7). (**A**) Molecular docking simulation of trypsin and the S10 protein. (**B**) Molecular docking simulation of trypsin and the S120 protein. (**C**) The number of intermolecular hydrogen bonds in the trypsin-S10 and trypsin-S120 systems changed with MD simulation time. (**D**) Changes in intermolecular hydrophobic interactions with MD simulation time in the trypsin-S10 and trypsin-S120 systems. (**E**) The change in intermolecular binding energy with simulation time in the trypsin-S10 and trypsin-S120 protein complex systems. (**F**) Superposition of the trypsin-S10 and trypsin-S120 complexes. The catalytically active amino acids Arg673 and Ser674 in the S protein exhibited obvious differences in the conformation of the two systems, and the side chain of Ser674 showed significant reversal.

To explore the mechanism underlying the difference in trypsin dependence between P10 and P120, sequence alignment analysis was performed on the P10 and P120 S genes, and the results revealed 16 amino acid site mutations (Table 2). Furthermore, based on the 3D structure file (6BFU) (18) and the sequence alignment file, structural similarity was inferred from the similarity in sequences. Homology modeling was performed for the P10 and P120 S proteins using the Modeler v9.19 program (19) to obtain a reasonable 3D structure model of the target protein, and the protein model was optimized by molecular mechanics. The crystal structure (4AN7) of trypsin was obtained from the Protein Data Bank (PDB) (20). Protein docking and MD simulation methods were used to investigate the molecular recognition of the trypsin and P10/P120 S proteins. Analysis of the protein docking results showed that the P10/P120 S proteins could bind to trypsin and that the binding region was located mainly near the catalytic site of trypsin (Fig. 6A and B). MD simulations showed that the trypsin-S120 system had stronger intermolecular hydrogen bonding and hydrophobic interactions than did the trypsin-S10 system (Fig. 6C and D). In addition, the binding energies of the trypsin-S10 and trypsin-S120 systems were basically stable, with average values of −1,009.09 ± 71.51 and −698.56 ± 70.54 kJ/mol, respectively (Fig. 6E). Furthermore, the analysis of the binding mode showed that the binding conformation of the S protein and trypsin changed significantly, mainly due to the mutation of S120 (Fig. 6F), and the above mutations led to a significant change in the conformation of the catalytically active amino acids, which may affect the catalytic effect of trypsin. In summary, the affinity of the S10 protein for trypsin was significantly greater than that of the S120 protein.

**Fig 7:**
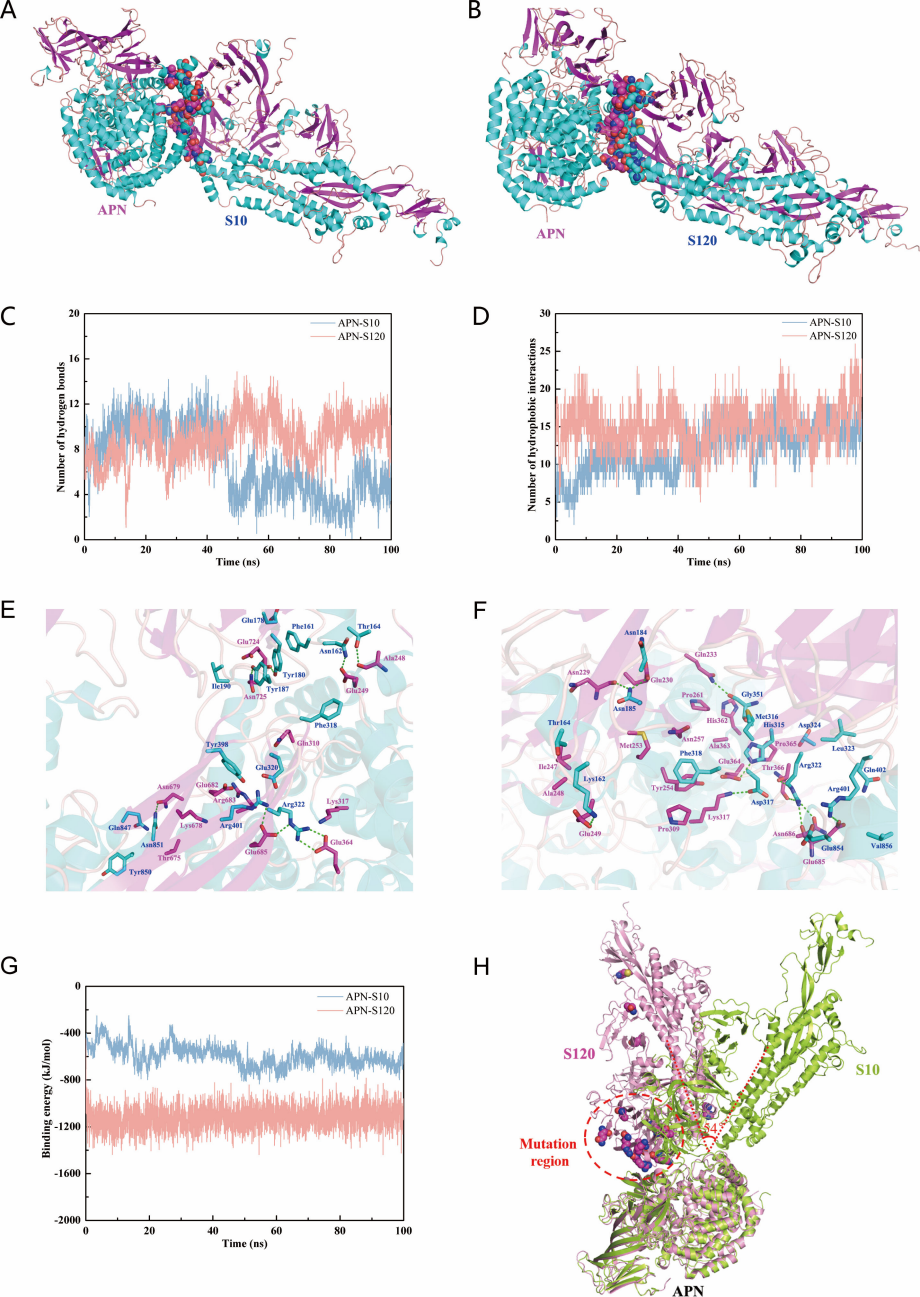
Molecular recognition analysis of the S10/S120 (PDB: 6BFU) protein with APN (7VPP). (**A**) Molecular docking simulation of the APN and S10 proteins. (**B**) Molecular docking simulation of APN and the S120 protein. (**C**) The number of intermolecular hydrogen bonds in the APN-S10 and APN-S120 systems changed with MD simulation time. (**D**) Changes in intermolecular hydrophobic interactions with MD simulation time in the APN-S10 and APN-S120 systems. (**E**) The three-dimensional interaction mode of APN and S10 (green dotted lines represent hydrogen bonds, and blue and purple represent amino acid residues in the APN and S10 proteins, respectively). (**F**) The three-dimensional interaction mode of APN and S120 (green dotted lines represent hydrogen bonds, and blue and purple represent amino acid residues in the APN and S120 proteins, respectively). (**G**) The change in intermolecular binding energy with simulation time in the APN-S10 and APN-S120 protein complex systems. (**H**) Superposition of the APN-S10 and APN-S120 complexes. The conformations of S10 and S120 binding to APN in the two systems are quite different, possibly because the mutation region in S120 is closer to the APN protein, resulting in an angle between S10 and S120 of approximately 54°.

Next, the crystal structure (7VPP) of aminopeptidase (APN) was obtained from the PDB (21). The molecular recognition of the APN and P10/P120 S proteins was analyzed by protein docking and MD simulation. The results showed that the P10/P120 S proteins could bind to the APN protein and that the binding region was mainly located at the binding position between APN and the S protein (Fig. 7A and B). MD simulations showed that the average hydrogen bond numbers of the APN-S10 and APN-S120 systems were 4.380 and 9.529, respectively, after 60 ns (Fig. 7C), indicating that the hydrogen bond interaction between the S120 protein and APN was stronger than that between the S10 protein and APN. In addition, the average number of hydrophobic interactions of APN-S10 and APN-S120 was 14.30 and 15.91, respectively (Fig. 7D), indicating that the hydrophobic interactions between the S120 protein and APN were slightly stronger than those between the S10 protein and APN. To further study the amino acid residues involved in the interaction between APN and the S protein, the protein binding pattern after MD simulation was analyzed. The results showed that S120 interacted with APN via more amino acid residues (Table 4) and a greater number of hydrogen bonds. Specifically, Ala248, Glu249, Glu364, Asn679, Glu685, Glu724, and Asn725 in APN formed hydrogen bonds with Asn162, Thr164, Tyr180, Tyr187, Arg322, Arg401, and Asn851 in the S10 protein, respectively, as did APN-Glu364 and S10-Arg322. There was also some degree of electrostatic salt bridge interaction between APN-Glu685 and S10-Arg322/Arg401 (Fig. 7E). Asn229, Glu230, Gln233, Glu249, Lys317, Glu364, Thr366, Gln685, and Asn686 in APN formed hydrogen bonds with Lys162, Asn185, His315, Asp317, Arg322, Gly351, and Arg401 in S120, respectively, as did APN-Glu249 and S120-Lys162. A stronger electrostatic salt bridge could be formed between APN-Lys317 and S120-Asp317 and between APN-Glu685 and S120-Arg322. In addition, the binding interface between APN and S129 contains several highly hydrophobic amino acid residues (e.g., Phe318, Tyr254, Met253, Ala363, Val856, and Leu323) that can form hydrophobic interactions and further enhance the affinity between the two (Fig. 7F). Different binding modes lead to differences in binding energies, and the binding energies of the APN-S10 and APN-S120 systems were basically stable after 60 ns, with average values of −629.06 ± 61.46 and −1,104.72 ± 90.25 kJ/mol, respectively (Fig. 7G).

**TABLE 4:** Important amino acid residue information involved in molecular recognition between APN and S10/S120

System	Interaction residues in APN	Interaction residues in S10/S120
APN-S10	Ala248, Glu249, Gly250, Gln310, Lys317, Glu364, Thr675, Lys678, Asn679, Glu682, Arg683, Glu685, Glu724, Asn725	Phe161, Asn162, Thr164, Glu178, Tyr180, Tyr187, Ile190, Phe318, Glu320, Arg322, Tyr398, Arg401, Gln847, Tyr850, Asn851
APN-S120	Asn229, Glu230, Gln233, Ile247, Ala248, Glu249, Met253, Tyr254, Asn257, Pro261, Pro309, Lys317, His362, Ala363, Glu364, Pro365, Thr366, Glu685, Asn686	Lys162, Thr164, Asn184, Asn185, His315, Met316, Asp317, Phe318, Arg322, Leu323, Asp324, Thr350, Gly351, Arg401. Gln402, Glu854, Val856

Furthermore, superposition analysis of the structures from the MD simulation was carried out separately. The results showed that the conformational binding between S10 and S120 and APN was quite different. The angle between S10 and S120 was approximately 54° (Fig. 7H), which was speculated to enhance the interaction between the S120 protein and APN due to the mutation of some amino acids, which in turn brought the mutation region closer to the APN protein and, thus, enhanced the interaction between the two. In conclusion, the affinity of the S120 protein for APN was significantly greater than that for S10 and APN (*P* > 0.05).

**Fig 8:**
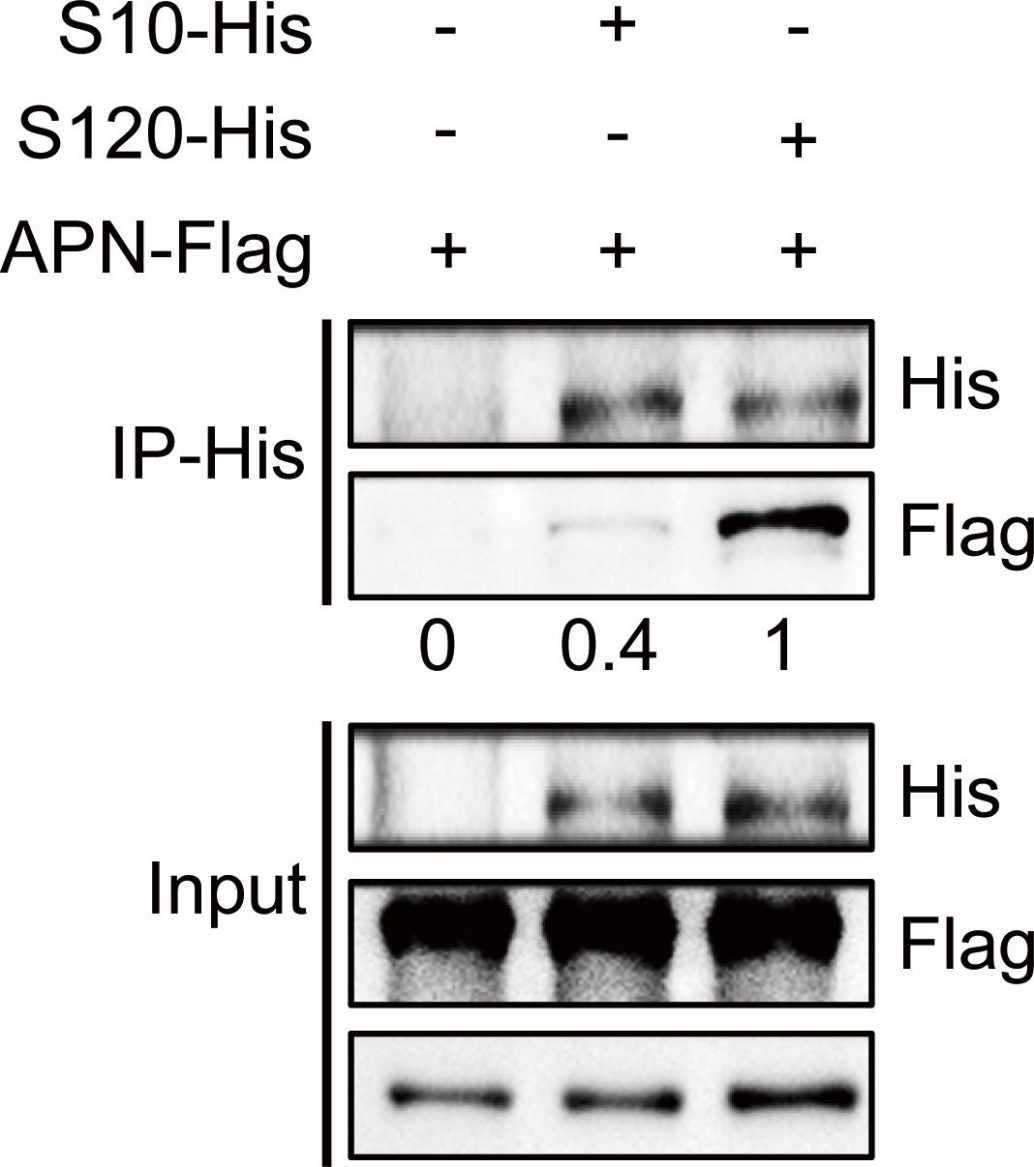
*In vitro*, the interaction between APN and S120 was stronger than that between APN and S10. The APN-Flag recombinant expression plasmid was cotransfected with S10 or S120 into 293T cells. After 24 h, the cell samples were collected and lysed, and His-tagged antibody was added. The APN protein was fished with S protein as bait protein and then detected by western blotting with His or Flag antibodies.

To further verify the difference in affinity between S10 and S120 for APN, the recombinant plasmid p3xFLAG-CMV-7.1-APN and pcDNA3.1-S10 or pcDNA3.1-S120 were cotransfected into 293T cells. After 24 h, the cell samples were collected for co-immunoprecipitation (co-IP). S10/S120 was used as bait for the fish APN protein (Fig. 8). The results showed that the APN band was more obvious in the S120 lane than in the S10 lane, indicating that the interaction between S120 and APN was stronger.

### P240 derived from serial passages could be applied as a live-attenuated vaccine to protect piglets against PDCoV challenge

To assess the protective effect of the P240-based live-attenuated vaccine, nine piglets from the immunization group and the challenged control group were orally administered virulent P6 (1 mL × 10^4.5^ TCID_50_/mL per piglet) at 28 days post-vaccination (dpv). The clinical symptoms and fecal RNA shedding of piglets in the immunization group and control group at 1–7 dpi after challenge with virulent P6 are shown in Table 5. In the immunization group, all five piglets were in good health during the experiment, and no clinical signs were observed (all FC scores equal to 0). Moreover, limited fecal RNA shedding was detected in the immunization group (all CT values greater than 30). In contrast, all four piglets in the challenged control group developed pasty and semifluid diarrhea (FC score: 0.50 ± 0.58–1.25 ± 0.50), and more viral RNA was detected in the feces at 2 dpi (CT values: 26.74 ± 0.91–29.97 ± 2.26). Finally, in the immunization group and the challenge control group, 5/5 and 0/4 of the piglets, respectively, were protected during the observation period. These data indicated that the P240-based live-attenuated vaccine could provide complete protection for piglets against challenges with virulent PDCoV and is a promising live-attenuated vaccine candidate.

**TABLE 5:** Clinical signs and fecal virus shedding of piglets after challenge with 1 mL of the virulent PDCoV CH/XJYN/2016-P6 (1 × 10^4.5^ TCID_50_/mL)^*a*^

dpi	Immunization group^*b*^ (*n* = 5)			Challenged control group^*c*^ (*n* = 4)		
	CT^*d*^	Average FC^*f*^	NP^*e*^	CT	Average FC	NP
0	31.90 ± 0.75	0	0/5	33.58 ± 1.36	0	0/4
1	34.50 ± 0.76			33.5 ± 0.22	0	0/4
2	34.10 ± 1.10			29.97 ± 2.26	0.75 ± 0.96	2/4
3	33.50 ± 1.59			28.61 ± 0.88	1.00 ± 1.15	2/4
4	35.49 ± 1.69			26.74 ± 0.91	1.25 ± 0.50	4/4
5	34.09 ± 0.48			29.70 ± 1.20	1.25 ± 0.50	2/4
6	32.92 ± 1.66			33.56 ± 0.32	0.50 ± 0.58	0/4
7	32.86 ± 1.31			33.07 ± 1.33	0.50 ± 0.58	0/4

^*a*^ CT value: the mean cycle threshold value; dpi: days post-inoculation; FC: fecal consistency; NP: number of PDCoV-positive pigs.

^*b*^ Piglets were intramuscularly injected with 1 mL of the PDCoV P240 (10^8.5^ TCID_50_/mL).

^*c*^ Piglets were intramuscularly injected with 1 mL of PBS.

^*d*^ A critical point was set at 30; CT values greater than 30 were considered negative.

^*e*^ Number of PDCoV-positive piglets.

^*f*^ Score: 0 = normal; 1 = pasty; 2 = semiliquid; 3 = liquid. A score greater than or equal to one was considered diarrhea.

## DISCUSSION

PDCoV has emerged as an enteric coronavirus in recent years, and there is currently no effective drug or commercial vaccine against it. In this study, the PDCoV CH/XJYN/2016 strain isolated and preserved in our laboratory was serially passaged in LLC-PK cells to P240. The genetic kinetics and pathogenicity changes during the passage process were analyzed to obtain a candidate PDCoV strain for a live vaccine. The molecular mechanisms involved in the attenuation process during passaging were also investigated.

Pathogenicity during serial passage *in vitro* (Table 1) and genetic evolution analysis of the genome (Tables 2 and 3) showed that with an increase in the number of passages, the adaptability of the PDCoV CH/XJYN/2016 strain in LLC-PK cells increased, and the pathogenicity in piglets gradually decreased, findings that are similar to those of a previous study that showed that PEDV was attenuated by passaging. For example, a pathogenicity study of PEDV PC22A strains passaged *in vitro* showed that the virulence of P100 and above was significantly reduced in 4-day-old piglets, cesarean section piglets, and piglets lacking colostrum and that P120 and P160 were attenuated in 4-day-old traditional piglets. Compared with that of P120, the replication efficiency of P160 in the pig intestine was lower (22). After 40-day-old weaned piglets were infected with the PEDV PC22A-P3 (virulent strain), PC22A-P100C4, or P120, P3 caused diarrhea in all pigs with a high level of fecal viral RNA shedding. P100C4 and P120 did not cause diarrhea in pigs, although viral RNA was detected in the feces of all pigs except for one inoculated with P100C4 (23). However, to date, there are no related reports on the complete attenuation of PDCoV strains. Therefore, in this study, for the first time, a completely attenuated PDCoV strain was developed through serial passaging *in vitro*.

Studies have shown that small but critical mutations in coronaviruses can significantly affect their pathogenicity, replication ability, and stability (24). In this study, genome sequence alignment of different generations of PDCoV (P0, P10, P20…P240) showed that PDCoV-P240 NSP3 had five amino acid mutations. NSP3 is the largest protein in coronaviruses and can act as a scaffold protein to interact with itself and bind to other viral nonstructural proteins or host proteins, playing an important role in the viral life cycle (25). PEDV attenuated the strain NSP3, which also had mutations in key amino acid sites (26). Taha et al. found that the amino acid mutation of SARS-CoV-2 NSP3 seriously affected its replication level in mice. These findings indicate that NSP3 is an important virulence gene of coronaviruses. Furthermore, the results from piglet vaccination and challenge experiments showed that the immunogenicity of attenuated P240 was maintained, indicating that these mutations obtained during serial passage may mainly contribute to reducing the pathogenicity of the virus. On the other hand, these mutations may be important because the infectivity of P240 significantly decreased after exposure to low pH.

The S gene of coronaviruses is associated with tissue tropism, host specificity, and genetic diversity (10, 27). In our study, the S gene of the PDCoV CH/XJYN/2016 strain had mutations during passaging from P0 to P240, resulting in a change of 26 amino acids. However, whether these mutations are associated with the reduced pathogenicity of PDCoV needs to be studied further. S gene sequence analysis from P0 to P240 showed that mutations from P20 to P60 were frequent; there were five mutations (N162K, N169K, N397K, Q489L, and N1029K) in P20, four of which were asparagine (N)-to-lysine (K) mutations. Asparagine is uncharged and has a weak interaction with surrounding amino acids or host proteins, and lysine is a charged basic amino acid that can interact with other charged amino acids to form salt bridges and strengthen tertiary structures. As in the SARS-CoV-2 study, S protein-specific N to K mutations seriously affect the conformational changes and binding activity of the protein to the receptor (28). As there are many mutations in the S protein, these mutations may act alone or through different combinations to change the function of the S protein. In this study, we aimed to identify a possible cause of these mutations, and the key functional sites of the S protein will be clarified in our follow-up study.

Previous studies have analyzed the global transcriptome signatures of host cells after infection with PEDV or PDCoV to gain insight into the response of host cells after viral infection (29–32). During the serial passage of the PDCoV CH/XJYN/2016 strain in LLC-PK cells, the susceptibility of the cells to PDCoV and the proliferation characteristics of the cells changed significantly. To study the mechanism underlying the biological differences of PDCoV passage strains in LLC-PK cells, transcriptome sequencing technology was used to analyze the differences in gene expression after infection with P0, P10, and P240. Compared to the NC cells, P10-infected cells had the most DEGs, while P240 had the fewest DEGs (Fig. 4A), which may be related to the fact that P240 gradually adapted to the cells with the continuous passage of PDCoV, which is consistent with the results of Peng et al. (33). GO and KEGG analyses revealed that compared with those in the NC group, differentially expressed genes were enriched in different cell functions and signaling pathways after different generations of PDCoV infection. The P0 infection group was enriched mainly in signaling pathways such as the immune response to the virus, while P10 and P240 were enriched mainly in important cell signaling pathways such as the TNF, Toll-like receptor, NOD-like receptor, RIG-I-like receptor, and P13K-Akt pathways (Fig. 4B and C). The differences in immune regulation induced by P0, P10, and P240 help us to understand their differences in pathogenicity and virulence, which are basically consistent with other studies on the kinetics of coronavirus infection, such as the broad-spectrum inhibition of the innate immune response by SARS-CoV-2 variants, suggesting that coronaviruses have a common strategy for immune evasion and viral proliferation (34).

The invasion of target cells by coronavirus depends on the hydrolysis of the S protein by various host cell proteases, such as furin-like protease, trypsin, TMPRSS2, and cathepsin L. Therefore, adding trypsin during *in vitro* culture can promote the replication of wild-type coronavirus strains and is widely used in the isolation of coronaviruses (7, 35, 36). Similarly, for PDCoV, trypsin is also essential for virus isolation and culture *in vitro* (7). This study revealed that during the serial passage of the PDCoV CH/XJYN/2016 strain, the trypsin dependence of the strain changed. Specifically, P10 could infect a large number of LLC-PK cells with obvious cytopathic effects with the addition of trypsin; without trypsin, the strain hardly infected LLC-PK cells with no cytopathic effects. P120 effectively infected LLC-PK cells in both the presence and absence of trypsin and had obvious cytopathic effects (Fig. 5). These results suggest that trypsin may have an important effect on the invasion of low-passage or wild-type PDCoV strains. However, studies by Yang et al. (37) showed that trypsin had no significant effect on the invasion or shedding of PDCoV but instead enhanced the spread of PDCoV among LLC-PK cells by promoting membrane fusion.

The coronavirus S protein is a type I membrane fusion glycoprotein. The process of recognition and membrane fusion with cell receptors requires the enzymatic cleavage of the host protease, which then undergoes large conformational changes to form a fused post-fusion conformation of the hairpin trimer. Moreover, the release of a large amount of energy promotes the occurrence of fusion events (38). In this study, by simulating the molecular recognition process of S10/S120 with trypsin or APN, it was found that the S10/S120 protein could bind to the trypsin protein, and the interaction between S10 and trypsin was stronger (Fig. 6). The S10/S120 protein can bind to the APN protein, and the interaction between S120 and APN is stronger (Fig. 7).

Further co-IP verification also confirmed this finding (Fig. 8). The results showed that the difference in the dependence of P10 and P120 on trypsin may be due to the mutation of the amino acid site of the S protein during viral passage and the conformation of the S protein changes, resulting in the interaction of the S protein with trypsin or the receptor (APN), thereby affecting the invasion of the virus into the cell. In particular, we confirmed that the S2 subunit could be a decisive factor in the dependence of coronavirus on trypsin, as has been reported in previous studies (39). Therefore, significant changes in the conformation of the S2 subunit caused by mutations could be another reason that the P120 virus was trypsin-independent.

As indicated by the results of the piglet challenge experiment, the P240-based live-attenuated vaccine could provide complete protection to piglets against virulent PDCoV challenge; these results are similar to those of previous studies of live-attenuated PEDV vaccines (40, 41). For example, the replication efficiency and infectivity of the attenuated PEDV virus in the intestine of piglets declined sharply, but its immunogenicity was maintained. As an attenuated live vaccine, intramuscular injection of PEDV provided complete protection to piglets and helped them resist the virulent PEDV virus (22, 40–43). In this study, limited viral shedding titers (Table 1) and no viral antigen distribution in the intestines (Fig. 2) suggested that P240 had limited infectivity in piglets compared with P10. Therefore, based on these results, we speculate that long-term passaging favors PDCoV adaptation to unnatural host cells (e.g., cell cultures) and results in an attenuated virus population with very limited infectivity in natural host tissues upon intestinal infection. Furthermore, mutation accumulation in the P240 population has likely reached a tolerable limit and has driven the viral population to the edge of survival in the piglet intestine (22, 43). Moreover, another possible reason for the reduced infectivity of P240 is that PDCoV resistance to acid was reduced following serial cell passage, which prevented high-passage PDCoV P240 from reaching and infecting intestinal epithelial cells through the stomach of piglets. However, these speculations need to be confirmed through further experiments using reverse genetics.

In addition, by comparing the currently confirmed PDCoV neutralizing and linear B-cell epitopes (S280-288 and N28-44) (44, 45) of P240 with those of P10, we found that these epitopes of the S and N proteins were highly homologous between P240 and P10, indicating that the epitopes of PDCoV did not significantly change during serial cell culture passaging *in vitro*. Furthermore, the levels of anti-PDCoV-specific IgG antibodies in piglets immunized with P240 by two intramuscular injections at 14 and 28 dpv were examined, and the results showed that the average OD values of the IgG titers were 0.7 and 1.0, respectively (data not shown), which were roughly equivalent to the average OD values of the IgG titers in piglets infected with virulent PDCoV in our previous piglet infection experiment (46). Thus, high-passage PDCoV P240 has good immunogenicity in piglets. In short, although P240 has limited infectivity in piglets, there was no significant change in its major antigen site, indicating that the immunogenicity of P240, when used as a live-attenuated vaccine, was not affected. Therefore, even though P240 almost completely lost infectivity in piglets, intramuscular injection still provided good protection to piglets against challenges with virulent PDCoV.

In summary, this study generated a PDCoV completely attenuated strain, PDCoV CH/XJYN/2016-P240, *in vitro* by serial passaging. In pathogenicity experiments using P240 in piglets, piglets had no diarrhea symptoms and no pathological damage to intestinal tissue, no PDCoV antigen was detected in the intestine, and limited fecal viral RNA excretion was detected, indicating that P240 was completely attenuated *in vitro*. Further analysis of the genomic genetic changes in the PDCoV passage strains revealed significant differences in the gene expression of P0, P10, and P240 in LLC-PK cells, the dependence of P10 and P120 on trypsin, the molecular recognition between S10 and S120 and trypsin or APN, and the resistance to pepsin and acid between P10 and P240, indicating that these differences may be related to the molecular mechanism of PDCoV CH/XJYN/2016 strain attenuation *in vitro*.

## MATERIALS AND METHODS

### Cells, viruses, and passages

LLC-PK cells were purchased from the American Type Culture Collection (CL-101) and cultured in minimum essential medium (MEM) supplemented with 10% fetal bovine serum, 1% NEAA, and 1% HEPES. The PDCoV CH/XJYN/2016 strain was isolated and stored in our laboratory (47). When the density of the LLC-PK cells reached approximately 80%, the culture medium was removed, and the cells were washed twice with Dulbecco's phosphate-buffered saline (DPBS). Then, the cells were inoculated with the P0 virus at an MOI of 0.1. P0 virus was diluted with 1 mL of MEM containing 1% NEAA, 1% HEPES, and 20 µg/mL trypsin, mixed evenly, added to a T25 culture flask, and incubated at a constant temperature of 37°C with 5% CO_2_ for 1 h. Supplementation with 4 mL of MEM containing 20 µg/mL trypsin was performed, cytopathy was observed every day, and the viral fluid was collected when the cytopathy reached 80%–90%. The viral fluid underwent three freeze (−80°C)-thaw cycles and was then aliquoted and stored in liquid nitrogen. The cryopreserved seed viruses were serially passaged as described above on LLC-PK cells up to passage 240.

### Pathogenicity of PDCoV serially passaged strains in 4-day-old piglets

Thirty-three 4-day-old conventional neonatal piglets with no history of PDCoV infection or vaccination were purchased from commercial pig farms. Before inoculation, piglet serum was collected and tested following the instructions of commercial kits to ensure that no PDCoV-specific antibodies were present. Additionally, RT-PCR using material collected from rectal swabs was negative for major porcine enteroviruses, such as PDCoV, PEDV, TGEV, and ProV. All piglets were randomly divided into seven groups (experimental group, five pigs/group; mock group, three pigs/group) and raised in separate cages and pens. The G1 group was orally administered 1 mL of MEM-treated P0 intestinal tissue grinding fluid. The G2–G6 groups were orally inoculated with P10, P50, P100, P160, and P240 at doses of 10^4.6^ TCID_50_/pig, 10^7.4^ TCID_50_/pig, 10^8.3^ TCID_50_/pig, 10^8.4^ TCID_50_/pig, and 10^8.5^ TCID_50_/pig, respectively. The mock group was administered 1 mL of MEM. During the experiment, clinical signs (diarrhea, vomiting, and anorexia) were monitored and recorded every day, and fecal consistency was scored as follows: solid (0 points), pasty (1 point), semifluid (mild diarrhea, 2 points), or liquid (severe diarrhea, 3 points). At the end of the experiment, intestinal tissue was collected for histopathological examination.

### Pepsin and acid tolerance test

To verify whether infectivity is related to pepsin tolerance, PDCoV P240 was treated with pepsin for 60 min and then used to infect LLC-PK cells at an MOI of 0.1, and cell samples were collected at 2, 6, 12, and 24 h for RT-qPCR detection. Furthermore, the influences of the gastric fluid of suckling piglets on the infectivity of PDCoV P10 and P240 were determined. A previous study showed that the pH of the gastric fluid of suckling piglets is approximately equal to 4.0 (48). Therefore, P10 and P240 were treated with MEM with a pH of 4.0 (low) or 7.2 (normal) for 60 min and then used to infect LLC-PK cells at an MOI of 0.1; cell samples were collected at 12 h for RT-qPCR.

### Growth curves

LLC-PK cells were inoculated with P10, P50, P100, P160, or P240 at an MOI of 0.001. Viral fluids were collected at 6, 12, 24, 36, 48, 60, 72, 84, and 96 h after infection. After three repeated freeze-thaw cycles, a viral 50% tissue culture infectious dose (TCID_50_/mL) was determined using the Reed–Muench method.

### RT-qPCR

Rectal swabs were collected from the piglets every day, treated with MEM, thoroughly vortexed, and then centrifuged at 3,500 rpm for 10 min. The supernatant was collected, and viral RNA was extracted. PDCoV RNA and fecal shedding titers were calculated using TaqMan real-time PCR. The sequences of the PDCoV N gene-specific primers and probes used were as follows: PDCoV-Q-forward: ACGTCGTAAGCCAGCATC; PDCoV-Q-reverse: CCCACCTGAAUGTTGCTCTC; and PDCoV-Q-probe: CY5-GTATGGCTGATCCTCGCATCATGGC-BHQ2. The thermal cycling parameters were 42°C for 5 min, 95°C for 10 s, 95°C for 5 s, and 57°C for 20 s for a total of 40 cycles.

### Indirect immunofluorescence assay

P0, P10, P50, P100, P160, or P240 at an MOI of 0.01 were used to inoculate LLC-PK cells in six-well plates. After 24 h of infection, the maintenance medium was discarded, and the cells were washed three times with DPBS. The cells were fixed with 4% paraformaldehyde at 4°C for 1 h, permeabilized with 0.25% Triton-100 for 10 min at room temperature, and then blocked with 5% BSA for 1 h. Mouse anti-PDCoV N protein monoclonal antibody and 488-labeled goat anti-mouse IgG were used as primary and secondary antibodies, respectively. Nuclei were stained with DAPI in the dark for 5 min. The cells were then washed three times with DPBS and observed under a fluorescence microscope.

### High-throughput sequencing of the whole genome of PDCoV serial passage strains

High-throughput sequencing (also known as second-generation sequencing and deep sequencing) technology can quickly and indiscriminately detect all nucleic acids in a sample through massively parallel sequencing. Viral RNA was extracted from PDCoV P0, P10, P50, P100, P160, and P240 cell cultures according to the instructions of the HiPure Viral RNA Kit. Nucleic acids were “translated” into data formats recognized by bioinformatics software through library construction and on-machine sequencing, and then, we obtained preliminary virus data after quality control of the software process, removal of rRNA, host sequences, bacteria, and other redundant sequences. Finally, the filtered data were assembled, aligned, annotated, and subjected to other analysis processes to obtain the genomic information of the virus quickly.

### Histopathology and immunohistochemistry

The jejunum of each piglet was collected at 7 dpi after the challenge and fixed with 4% paraformaldehyde solution. The fixed tissues were dehydrated, trimmed, embedded, sectioned, stained with hematoxylin, and mounted following standard pathological examination procedures before being observed under a microscope. The PDCoV N protein mAb prepared and stored in our laboratory was used as the primary antibody, and horseradish peroxidase (HRP)-labeled goat anti-mouse IgG was used as the secondary antibody. Images of the sections were acquired using a Pannoramic 250 digital slide scanner and a BA200 digital trinocular camera microphotography system.

### Molecular analysis of the S protein and trypsin

The crystal structures in the PDB were retrieved by the protein BLAST tool of NCBI. The Modeler v9.19 program was used for the homology modeling of the S protein to obtain a reasonable 3D model of the target protein, and the protein model was subjected to molecular mechanics optimization. The crystal structures of the S protein, trypsin, and APN were obtained from the PDB. The crystal structure was a complex of trypsin and the spike protein fragment and was used as a reference for the binding of the S protein to trypsin or APN. Molecular docking and molecular dynamics simulation methods were used to study the molecular recognition process of trypsin or APN for the S protein S10/S120, respectively.

PDCoV S10, S120, and APN were cloned and inserted into eukaryotic expression vectors to obtain the recombinant plasmids pcDNA3.1-S10, pcDNA3.1-S120, and p3xFLAG-CMV-7.1-APN. The recombinant plasmids p3xFLAG-CMV-7.1-APN and pcDNA3.1-S10 or pcDNA3.1-S120 were cotransfected into 293T cells. After 24 h, the cells were lysed using an IP cell lysis buffer containing a mixture of protease inhibitors (P0013, Beyotime). The lysates were collected and incubated with 2 µg of anti-6× His tag antibody (Ab9108, Abcam) overnight at 4°C. The next day, 40 µL of Protein A + G Agarose (P2012, Beyotime) was incubated at 4°C on a shaker for 4 h, washed 3–4 times with 1× PBS, and resuspended in lysis buffer. Specific antibodies were used for immunoblotting to analyze proteins.

### Transcriptome sequencing analysis

LLC-PK cell monolayers were infected with P0, P10, and P240 viruses (MOI = 0.1). Cell samples were collected after 24 h, RNA was extracted for transcriptome-sequencing analysis, and a NC group was established. Eukaryotic mRNA was enriched using oligo (dT) magnetic beads to synthesize double-stranded DNA, which was subjected to end repair, the addition of poly(A), sequencing adapters, purification, and fragment selection using magnetic beads, and ultimately, libraries were obtained from PCR amplification. After the library was approved for sequencing, the raw reads were subjected to quality control (QC) to determine whether the sequencing data were suitable for subsequent analysis. After QC, filtered clean reads were aligned to the reference sequences, and the distribution and coverage of the reads in the reference sequences were counted to determine whether the alignment results passed the second QC (QC of alignment). After passing QC, a series of subsequent analyses, including gene expression, alternative splicing, novel transcript prediction, SNP detection, and gene structure optimization, were performed, and DEGs between samples were screened from the gene expression results. Based on the DEGs, GO functional enrichment analysis and KEGG pathway enrichment analysis were performed.

### Piglet vaccination and challenge experiment

Nine 4-day-old PDCoV-naïve piglets that tested seronegative for PDCoV by enzyme linked immunosorbent assay (ELISA) were obtained from a commercial pig farm with no previous history of PDCoV outbreak or PDCoV vaccination. All nine piglets were randomly divided into an immunization group (*n* = 5) and a challenge control group (*n* = 4). Each group was housed in separate rooms for the duration of the experiment. Piglets in the immunization group and the challenge control group were immunized intramuscularly at 0 dpv with 1 mL of the prepared experimental P240-based live-attenuated vaccine (viral antigen concentration: 10^8.5^ TCID_50_/mL) and PBS, respectively. All piglets received booster immunizations at 14 dpv with the same dose of vaccine or volume of PBS. At 28 dpv, all piglets were challenged orally with 1 mL of virulent PDCoV CH/XJYN/2016-P6 (1 mL × 10^4.5^ TCID_50_/mL). After the challenge, clinical signs of PDCoV infection were observed daily for 7 days, and clinical scores of FC were determined as follows: 0 = normal; 1 = pasty; 2 = semiliquid; and 3 = liquid. Fecal samples were collected daily from all piglets and tested by real-time PCR for the duration of the challenge to monitor viral shedding in feces. CT values greater than 30 were considered negative. Piglets were identified as PDCoV infection-positive when the FC score was ≥1 and viral shedding was detected in the fecal samples at the same time.

### Data analysis

All the statistical analyses were performed using GraphPad Prism software. Statistical significance was assessed using Fisher’s exact test or the chi-squared test (asterisk * indicates statistical significance: no significant difference at *P* > 0.05, no significant difference at ^*^P).

## Data Availability

The data supporting the results of this study are available from the corresponding author upon reasonable request.
